# Distinct structural and functional angiogenic responses are induced by different mechanical stimuli

**DOI:** 10.1111/micc.12677

**Published:** 2021-01-23

**Authors:** Roger W. P. Kissane, Peter G. Tickle, Natalie E. Doody, Abdullah A. Al‐Shammari, Stuart Egginton

**Affiliations:** ^1^ Department of Musculoskeletal & Ageing Science University of Liverpool Liverpool UK; ^2^ School of Biomedical Sciences University of Leeds Leeds UK; ^3^ Department of Mathematics, Faculty of Sciences Kuwait University Khaldiya Kuwait; ^4^ Department of Genetics & Bioinformatics Dasman Diabetes Institute Dasman Kuwait

**Keywords:** capillary supply, mathematical modelling, overload, shear stress, skeletal muscle

## Abstract

**Objective:**

Adequacy of the microcirculation is essential for maintaining repetitive skeletal muscle function while avoiding fatigue. It is unclear, however, whether capillary remodelling after different angiogenic stimuli is comparable in terms of vessel distribution and consequent functional adaptations. We determined the physiological consequences of two distinct mechanotransductive stimuli: (1) overload‐mediated abluminal stretch (OV); (2) vasodilator‐induced shear stress (prazosin, PR).

**Methods:**

*In situ* EDL fatigue resistance was determined after 7 or 14 days of intervention, in addition to measurements of femoral artery flow. Microvascular composition (muscle histology) and oxidative capacity (citrate synthase activity) were quantified, and muscle PO_2_ calculated using advanced mathematical modelling.

**Results:**

Compared to controls, capillary‐to‐fiber ratio was higher after OV14 (134%, *p* < .001) and PR14 (121%, *p* < .05), although fatigue resistance only improved after overload (7 days: 135%, 14 days: 125%, *p* < .05). In addition, muscle overload improved local capillary supply indices and reduced CS activity, while prazosin treatment failed to alter either index of aerobic capacity.

**Conclusion:**

Targeted capillary growth in response to abluminal stretch is a potent driver of improved muscle fatigue resistance, while shear stress‐driven angiogenesis has no beneficial effect on muscle function. In terms of capillarity, more is not necessarily better.

**Abbreviations**
C:F: capillary‐to‐fiber ratio
CD: capillary density
COPD: chronic obstructive pulmonary disease
CS: citrate synthase
CSA: cross‐sectional area
EDL: extensor digitorum longus
M_b_
: body mass
MO_2_
: oxygen demand
OV14: 14 days overload
OV7: 7 days overload
PO_2_
: partial pressure of oxygen
PR14: 14 days prazosin
PR7: 7 days prazosin
TA: tibialis anterior
VEGF: vascular endothelial growth factor

## 1 INTRODUCTION

Angiogenesis is a complex, highly coordinated process whereby an interplay between metabolic and mechanical stimuli promotes expansion of an existing microvascular network.^1^ This process is an essential component of postnatal adaptive remodelling, providing the microvascular capacity to support adequate tissue oxygenation. Clinical observations and experimental approaches highlight the importance of maintaining a healthy microcirculation in skeletal muscle, where expansion of the capillary bed accompanies improved fatigue resistance,^2^, ^3^, ^4^ while capillary rarefaction is associated with functional impairment.^4^, ^5^, ^6^, ^7^, ^8^, ^9^, ^10^ Further, the distribution of capillaries within muscle has a significant bearing on the functional capacity, as increasing heterogeneity of capillary spacing is predicted to reduce tissue oxygenation, hence compromising muscle endurance.^11^, ^12^, ^13^ Consequently, interactions between different levels of organization may determine functional capacity, for example, those involving adequate capillary density, optimal vessel spacing, and efficient tissue oxygenation.

Multiple, potentially synergistic angiogenic signalling pathways regulate the onset and cessation of capillary growth.^14^ The drivers for adaptive remodelling can be complex, for example, angiogenesis as a result of exercise training or indirect electrical stimulation^15^ may involve functional hyperemia, as well as metabolic or mechanical signals.^1^ While distinct morphological differences exist in formation of new capillaries induced by elevated shear stress (splitting angiogenesis) and canonical capillary growth (sprouting angiogenesis), upregulation of VEGF is common to both pathways.^16^, ^17^, ^18^ The capillary network of skeletal muscle is markedly expanded following imposition of mechanical overload,^17^, ^19^, ^20^, ^21^ with consequent improvement in fatigue resistance.^4^ Angiogenesis in response to mechanotransduction of the imposed stretch involves proliferation of endothelial cells and basement membrane degradation after upregulation of pro‐angiogenic VEGF and its principal signalling receptor Flk1, allowing sprouting filopodia to direct vessel formation.^16^, ^18^, ^20^, ^22^ No significant changes in muscle fiber type, oxidative capacity or arterial blood flow are observed in this process,^4^, ^23^, ^24^ indicating that microvascular proliferation is the key determinant of enhanced fatigue resistance. This primarily mechanically mediated angiogenic stimulus contrasts with the effects of active training whereby an interacting combination of functional hyperemia, metabolic and mechanical factors induce angiogenesis and remodelling in skeletal muscle.^14^

Chronic administration of vasodilators leads to an expansion of the microvascular system within skeletal muscle.^25^, ^26^ Angiogenesis induced by prazosin, an α_1_‐blocker, is nitric oxide dependent and driven by an increase in vascular shear stress.^16^, ^17^, ^25^ Fine structural changes in capillaries allow for longitudinal divisions of vessel lumen to produce new functioning capillaries, with an orientation of daughter vessels in the same direction as existing vessels.^22^ These morphological changes are maintained by continued exposure to the hyperemic stimulus, but are reversible as vessel rarefaction rapidly follows cessation of treatment.^25^ Given the low metabolic cost of this form of mechanotransduction‐induced angiogenesis, involving a low cellular turnover rate, it is considered an efficient form of microvascular expansion.^22^, ^25^ Nevertheless, as there is little evidence for any change in muscle phenotype (and hence altered metabolic feedback), prazosin‐induced angiogenesis has been assumed to result in “luxury” tissue perfusion, that is, supra‐normal capillarity, with hitherto equivocal functional implications.^27^

Better understanding of the differential response to overload‐induced longitudinal stretch and prazosin‐mediated shear stress may be important for developing effective angiotherapies designed to alleviate symptoms of chronic diseases involving a compromised microcirculation such as coronary artery disease, peripheral artery disease, diabetes etc. While attempts to provide direct pro‐angiogenic treatments *via* modulation of the VEGF signaling cascade have produced mixed results,^28^ established models of angiogenesis (muscle strain‐ or shear stress‐driven) may be utilized to quantify the efficacy of capillary growth in response to more integrative stimuli. It is notable that the functional consequences of these distinct vascular growth processes are poorly understood and only limited data is available on the interplay between capillarization, muscle performance and blood flow,^4^ confounding comparisons of the relative efficacy of potential angiogenic therapies for restoring impaired muscle performance. We hypothesized that the endurance (fatigue resistance) of skeletal muscle is dependent upon the mechanism driving adaptive angiogenesis (new vessel growth). By quantifying the effect of different mechanical stimuli—muscle stretch (overload) or elevated shear stress (prazosin administration)—on histological, biochemical and functional determinants of muscle performance, we demonstrate that angiogenesis drives improvements only after provision of an appropriately targeted stimulus working in concert with relevant local tissue feedback (ie, stimuli associated with increased muscle activity; localized metabolic signals and tissue oxygen status).

## 2 MATERIAL AND METHODS

### 2.1 Ethics

All procedures were approved by University of Leeds Animal Welfare and Ethics Committee and were performed in accordance with UK Animals (Scientific Procedures) Act 1986.

### 2.2 Animals

Male Wistar rats were housed on a 12 h light/dark cycle with *ad libitum* access to food and water and randomly assigned to one of five groups: control (CT; N = 10; *M*_b_ = 245 ± 25 g); muscle overload for seven (OV7; N = 10; *M*_b_ = 254 ± 14 g) or 14 days (OV14; N = 5; *M*_b_ = 271 ± 17 g); prazosin treatment for seven (PR7; N = 10; *M*_b_ = 285 ± 23 g) or 14 days (PR14; N = 5 *M*_b_ = 272 ± 25 g).

### 2.3 Overload surgery

Surgical anesthesia was induced and maintained using isoflurane (5% and 2%, respectively, in 100% O_2_; IsoFlo^®^). An incision was made along the distal two thirds of the TA, toward the lateral side of the muscle. Covering facia was cleared to expose the TA, allowing surgical release of the tendon and dissection from the lateral surface of the tibia. This procedure induces a functional overload leading to hypertrophy of the synergist EDL, an angiogenic response mediated *via* upregulation of VEGF and Flk‐1,^18^, ^19^ and altered metabolic profile.^21^, ^24^ Post‐operative analgesia (buprenorphine: 0.015 mg kg^−1^; Vetergesic^®^, Ceva, Amersham, UK) and antibiotic (Enrofloxacin: 2.5 mg kg^−1^; Baytril^®^, Bayer, Reading, UK) were provided for two days.

### 2.4 Prazosin supplementation

Prazosin was provided by *ad libitum* access to supplemented drinking water. This solution was made up by dissolving prazosin hydrochloride powder (Alfa Aesar, Ward Hill, MA, USA) at 40°C (50 mg L^−1^) in water sweetened to improve palatability.

### 2.5 In situ experiments

Following induction of anesthesia using isoflurane (4% in 100% O_2_), the descending jugular vein was catheterized to allow constant infusion (30–35 mg kg^−1^ h^−1^) of alfaxalone (Alfaxan, Jurox, Crawley, UK) to maintain the surgical plane. A catheter connected to a pressure transducer (AD Instruments, UK) was implanted in the carotid artery to facilitate monitoring of blood pressure and heart rate throughout the experiment, thereby ensuring that normal cardiovascular condition was maintained. A tracheotomy was also performed to allow spontaneous breathing.

### 2.6 Muscle performance and hindlimb blood flow

If not in an overload group, the intact TA was removed to allow unimpeded access to the EDL. Muscle force was quantified by attaching the distal tendon of the EDL to an ergometer lever arm (305B‐LR, Aurora Scientific Inc., Ontario, Canada). The peroneal (lateral popliteal) nerve was exposed and indirectly stimulated using stainless steel electrodes sutured parallel to the nerve axons. Muscle length and current delivery were optimized to generate maximal isometric twitch force before the onset of fatigue experiments. The EDL were fatigued by a protocol that applied bilateral 10 Hz stimulation (pulse width 0.3 ms, supramaximal voltage) for three minutes. A fatigue index (FI) was calculated as final tension/peak tension × 100, using five consecutive twitches in each case.

The proximal femoral artery was freed from surrounding tissue to enable application of a perivascular flow probe (0.7PSB, Transonic, Ithaca, NY). This enabled simultaneous measurement of arterial blood flow and development of muscle force. Blood flow is presented at two timepoints: resting (ie, before onset of fatigue protocol) and end‐stimulation (ie, immediately before the cessation of stimulation).

### 2.7 Tissue preparation

Animals used exclusively for histology were culled through concussion and cervical dislocation, while animals under terminal anesthesia for *in situ* experiments were euthanized through cervical dislocation. The EDL muscles were carefully dissected and divided into three pieces. Proximal and distal portions were snap frozen in liquid nitrogen for enzyme assays, while the muscle mid‐belly was snap frozen in liquid nitrogen cooled isopentane for muscle histology. All frozen tissue was stored at −80°C until point of assay or sectioning. Serial cryostat sections (10 µm) were cut at −20°C and attached to polysine coated slides (VWR International) and stored at −20°C until staining.

### 2.8 Citrate synthase assay

Snap frozen proximal and distal portions of the EDL (~30 mg) were homogenized in a reaction medium (100 mM Tris Buffer (pH 8), 100 μM 5,5ʹ‐dithiobis (2‐ nitrobenzoic acid), 300 μM acetyl‐CoA) using 7 mm ball bearings in a bead mill (Qiagen, TissueLyser LT). CS activity was measured through a change in optical density at 412 nm,^29^ measurements were taken from the most linear portion of the curve.

### 2.9 Immunohistochemistry, morphometric analysis and PO_2_ modelling

Capillaries were identified using *Griffonia simplicifolia* lectin‐1 (Vector Laboratories, UK) due to its binding affinity to proteoglycans in the glycocalyx of the microvasculature. Four fields of interest (each 0.145 mm^2^) were taken across the heterogeneous EDL.^30^ Global morphometric indices for capillary supply were calculated (capillary‐to‐fiber ratio, C:F; capillary density, CD). Individual capillary locations were digitized to generate a binary distribution and used to calculate local capillary supply regions and model tissue PO_2_.^31^ Briefly, the local geometric tissue supply regions (capillary domain area, CDA) were calculated as the area closest to an individual capillary, with the domain border placed equidistant between adjacent capillaries. Spatial heterogeneity of the domain area follows a log‐normal distribution, and an appropriate index of variance is provided by the standard deviation of the logarithm of domain areas (LogSD). Tissue PO_2_ and MO_2_ were calculated using the Oxygen Transport Modeler.^31^ Briefly, tissue oxygen consumption was modelled *via* Michaelis‐Menton kinetics,^10^ using histology derived distributions of capillaries and typical skeletal muscle oxygen demand values.^32^, ^33^ We also modelled tissue PO_2_ using MO_2_ values derived from CS activity.^9^

### 2.10 Statistical analysis

All data are expressed as mean ± standard deviation. Individual numbers of animals for each group are presented within figure legends (*n*). Significant differences (*p* < .05*)* between groups were determined by one‐way analysis of variance (ANOVA) with Tukey post‐hoc tests in SPSS (v.25).

## 3 RESULTS

### 3.1 Longitudinal stretch and shear stress are potent drivers of skeletal muscle angiogenesis

**FIGURE 1:**
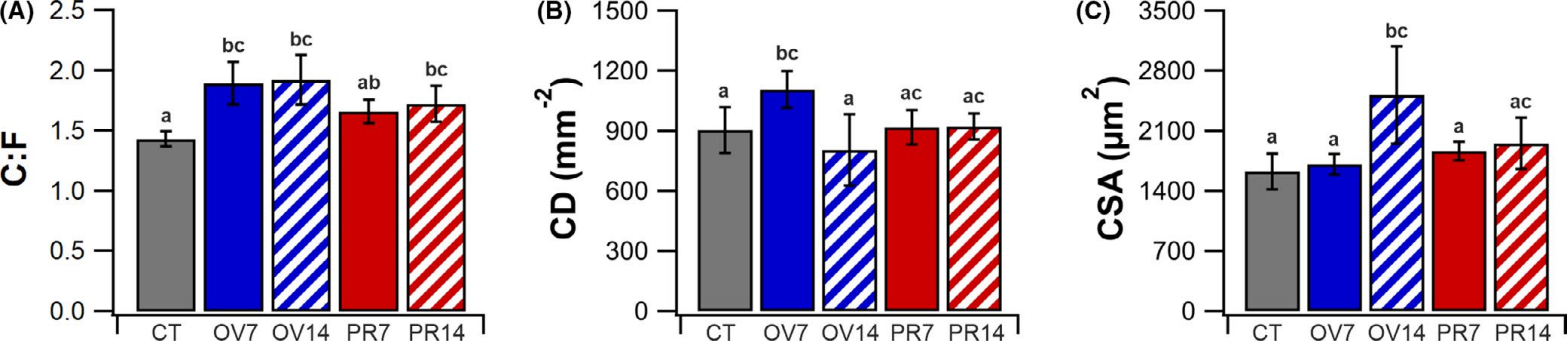
Angiogenic response to longitudinal stretch and increased luminal shear stress. Change in capillary‐to‐fiber ratio (C:F) of the EDL (A), capillary density (CD) (B) and fiber cross‐sectional area (CSA) (C). Mean ± SD, unmatched lower‐case letters denote statistical significance (*p* < .05). CT (*6*), OV7 (*5*), OV14 (*4*), PR7 (*5*) and PR14 (*4*)

**FIGURE 2:**
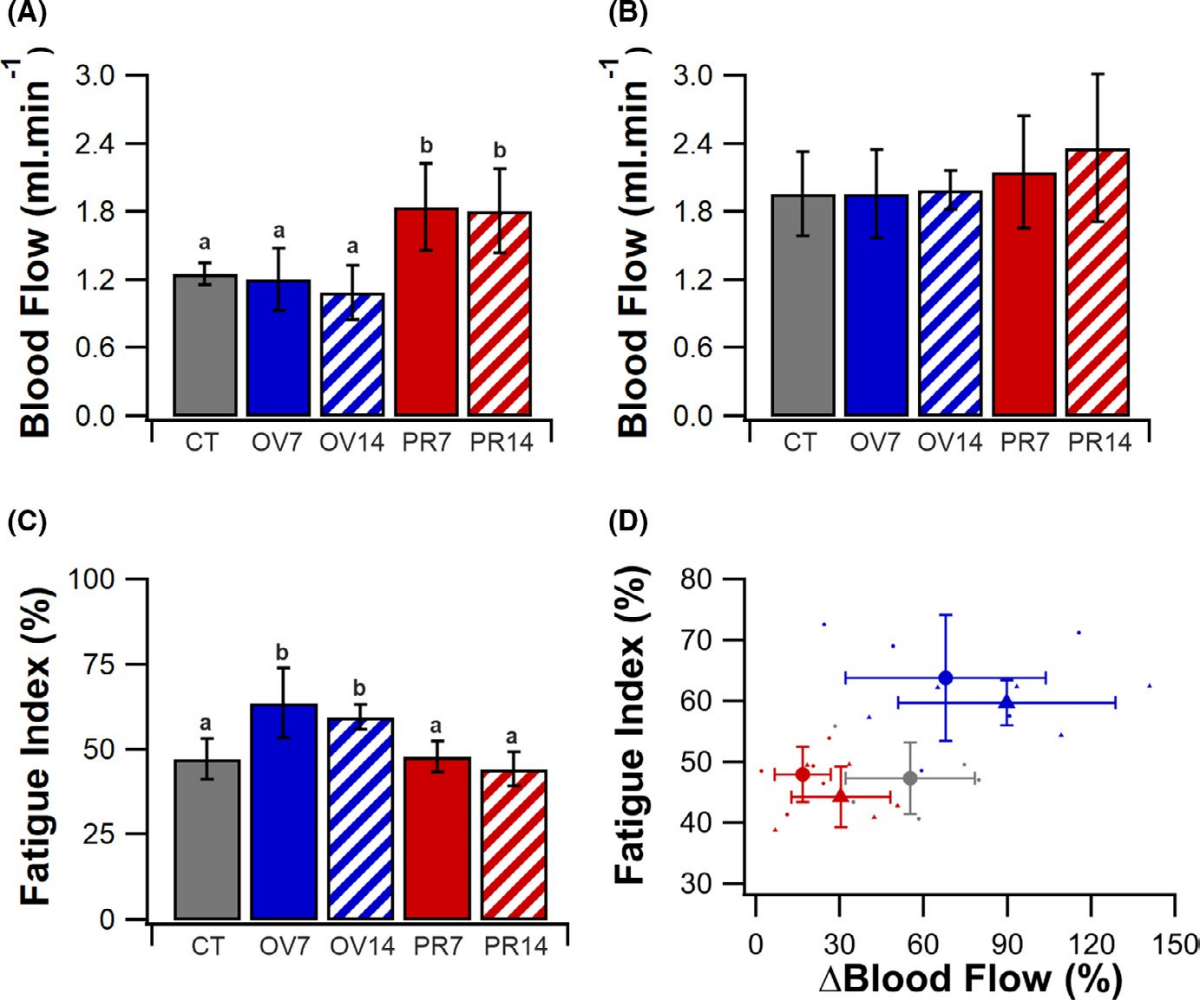
*In situ* arterial blood flow and muscle fatigue resistance. Hindlimb blood flow at rest (A) and immediately following three minutes of stimulation. (B) EDL fatigue index following three minutes of indirect electrical stimulation at 10 Hz. (C) Scatter plot presenting individuals fatigue index against functional hyperemia (percentage increase in femoral blood flow from rest). (D) Scatter data presented as control (gray circles), OV7 (blue circles) OV14 (blue triangles), PR7 (red circle) and PR14 (red triangles). Mean ± SD, unmatched lower‐case letters denote statistical significance (*p* < .05). CT (*5*), OV7 (*5*), OV14 (*5*), PR7 (*5*) and PR14 (*5*)

Both muscle overload and prazosin supplementation significantly increased capillary content within the EDL following 14 days of treatment (Figure 1). C:F was higher in OV7 (1.90 ± 0.18, *p* < .05) and OV14 (1.92 ± 0.10, *p* < .05) compared to CT (1.43 ± 0.06), while prazosin supplementation led to a higher C:F after 14 days (1.73 ± 0.15, *p* < .05). Using an area‐based descriptor of vascular supply, CD (Figure 1B), the angiogenic response to muscle overload presents as a dynamic event, significantly elevated at seven days (1108 ± 91 mm^−2^, *p* < .05) compared to control (904 ± 115 mm^−2^), returning toward control levels at 14 days (804 ± 179 mm^−2^). The adjustment in CD at 14 days is due to a significant hypertrophy of muscle fibers in response to muscle overload (2522 ± 567 µm^2^ *vs*. 1629 ± 210 µm^2^, *p* < .05). In contrast, both seven and 14 days of prazosin supplementation failed to significantly elevate CD (Figure 2B), partly due to the larger fiber CSA of the animals at seven and 14 days (Figure 1C).

### 3.2 Global supply does not predict functional outcomes

Resting hindlimb blood flow was unchanged in OV7 (*p* = .998) and OV14 (*p* = .889) (Figure 2A), and was similar to CT (1.26 ± 0.10 ml·min^−1^). Prazosin supplementation resulted in a higher resting blood flow in PR7 (1.84 ± 0.39 ml·min^−1^, *p* < .05) and PR14 (1.81 ± 0.37 ml·min^−1^, *p* < .05). Hindlimb blood flow immediately following the fatigue protocol did not differ significantly across the five experimental groups (Figure 2B); however, the control (56%, *p* < .05) OV7 (63%, *p* < .05) and OV14 (83%, *p* < .05) were all significantly increased from baseline, while PR7 (17%, *p* = .97) and PR14 (31%, *p* = .134) were not. Overload improved muscle fatigue resistance compared to CT at 7 (*p* = .004) and 14 (*p* = .041) days, with a 35 and 26% increase in FI, respectively (Figure 2C). However, despite a significant increase in microvascular content (Figure 1A) there was no effect on muscle fatigue resistance in PR7 (*p* = 1) or PR14 (*p* = .938) (Figure 2C). It appears, there may be a relationship between the available functional hyperemia response and the resistance to fatigue (Figure 2D); however, there is no significant relationship in the overload (R^2^ = .01, *p* = .779) or prazosin (R^2^ = .02, *p* = .892) animals.

### 3.3 Functional capacity is associated with changes in local distribution of capillaries

**FIGURE 3:**
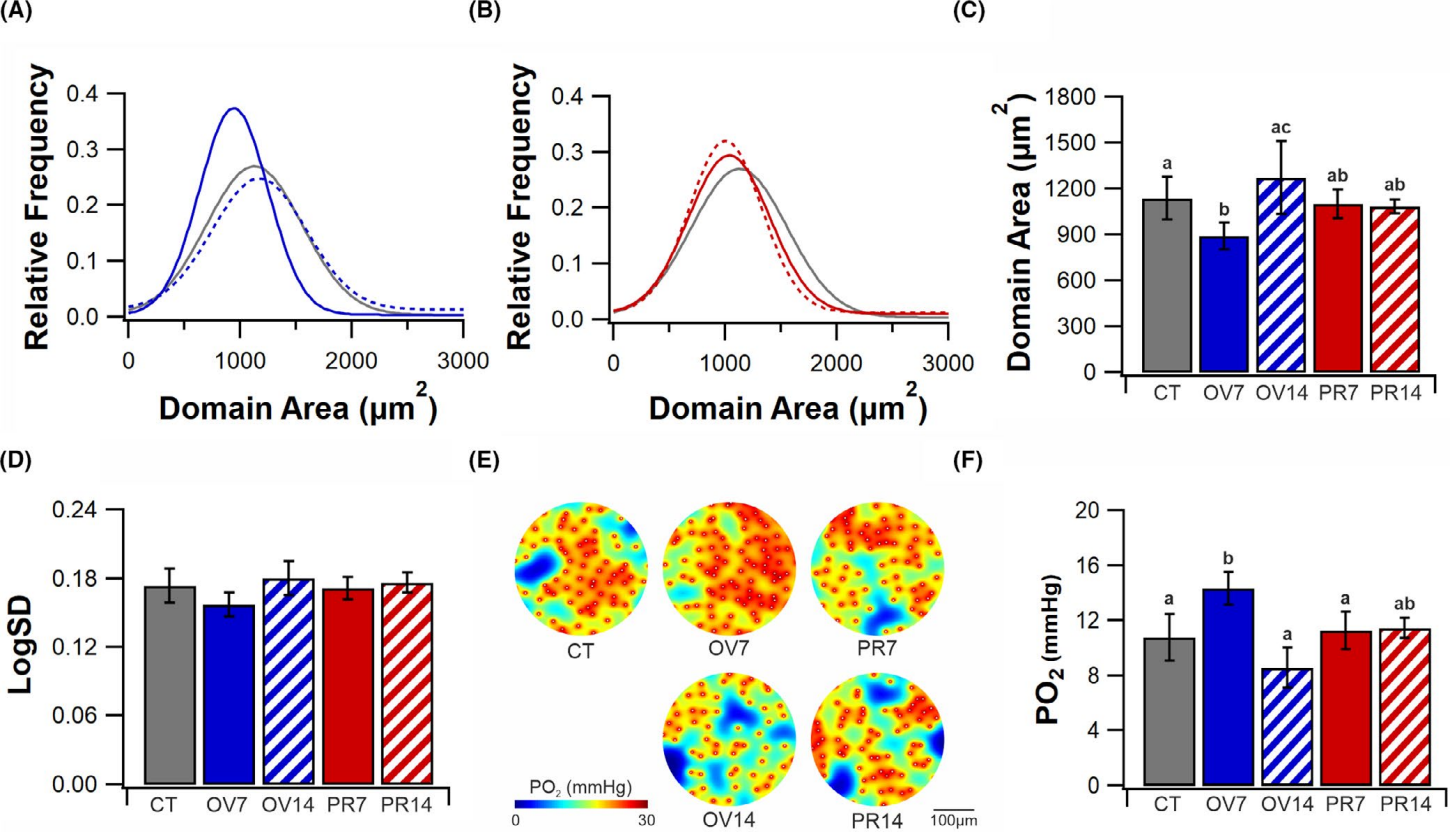
Local capillary supply. Change in distribution of capillary domain area following seven (solid line) and 14 days (dashed line) of overload (A, blue) and prazosin (B, red) *vs*. control (gray). Average capillary domain area (C) and LogSD. (D) Histological based mathematical modelling of PO_2_ (E) and average tissue PO_2_ (F). Mean ± SD, unmatched lower‐case letters denote statistical significance (*p* < .05). CT (*5*), OV7 (*5*), OV14 (*4*), PR7 (*5*) and PR14 (*4*)

Capillary domain area and heterogeneity of supply are effective predictors of tissue PO_2_ (Figure 3). Capillary domain area was smaller in OV7 compared to CT (893 ± 86 µm^2^ vs. 1138 ± 141 µm^2^, *p* < .05, Figure 3C) with a 10% decrease in LogSD (0.158 ± 0.010 vs. 0.174 ± 0.015, *p* = .251, Figure 3D). Local tissue remodelling of capillary supply was linked with a greater capacity to deliver and utilize O_2_ (Figure 3E‐F). In contrast, hypertrophy of muscle fibers in OV14 (Figure 1C) lead to normalization of capillary supply area (Figure 3A,C,D) characteristic of a further dynamic response, that may be associated with a slight reduction in fatigue resistance. Conversely, neither PR7 or PR14 had any significant change in average capillary domain area (Figure 3C) or distribution of capillaries (Figure 3D). Mathematical modelling of tissue PO_2_ indicated that capacity to deliver or utilize O_2_ was unaffected by prazosin treatment (Figure 3E‐F).

### 3.4 The differential response of muscle oxidative demand to longitudinal stretch and elevated shear stress

**FIGURE 4:**
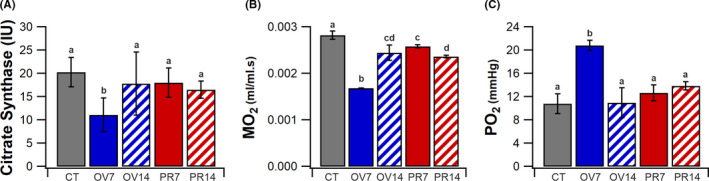
Adjustments in metabolic profile. Changes in citrate synthase (CS) activity (A) and consequence on calculated muscle MO_2_. (B) Effect of altered tissue MO_2_ on whole tissue PO_2_. (C) Mean ± SD, unmatched lower‐case letters denote statistical significance (*p* < .05). CT (*5*), OV7 (*5*), OV14 (*4*), PR7 (*5*) and PR14 (*4*)

A dynamic shift in muscle metabolic profile follows overload, but is not present with prazosin treatment. Decreased CS activity (20.27 ± 3.15 vs. 11.06 ± 3.63 IU, *p* < .05, Figure 4A) and reduced calculated muscle oxidative demand (0.0028 ± 0.0009 *vs*. 0.0017 ± 0.0001 ml.ml^−1^·s^−1^, *p* < .05, Figure 4B) was observed in OV7, which resulted in reciprocally higher tissue PO_2_ compared to CT (10.8 ± 1.7 vs. 20.8 ± 0.9 mmHg, *p* < .05, Figure 4C). This metabolic shift was almost completely attenuated in OV14 with a restoration of CS activity (17.78 ± 6.80 vs. 20.27 ± 3.15 IU, *p* = .908, Figure 4A), although calculated tissue oxygen demand still remained lower than CT (0.0025 ± 0.0002 vs. 0.0028 ± 0.0009 ml.ml^−1^·s^−1^, *p* < .05, Figure 4B). CS activity was not different from CT in PR7 (*p* = .936) and PR14 (*p* = .743, Figure 4A), despite evident decrease in modelled muscle oxygen demand (Figure 4B), the tissue oxygenation profile (Figure 4C) was similar to CT in PR7 (*p* = .352) and PR14 (*p* = .055).

## 4 DISCUSSION

**FIGURE 5:**
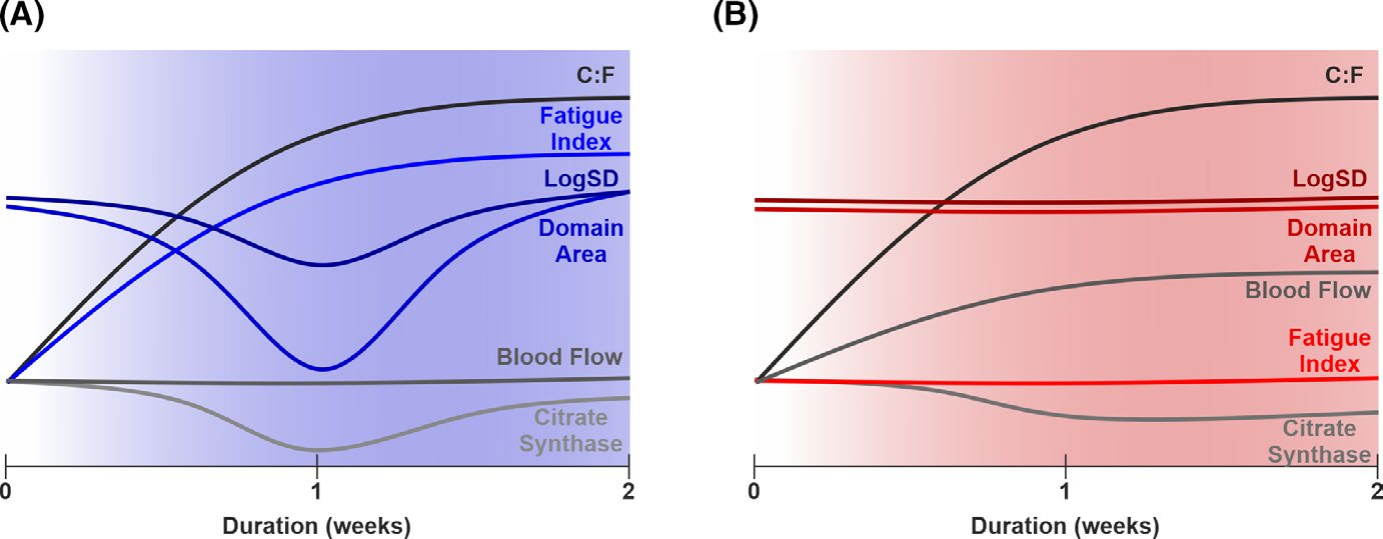
Overview of the dynamic response to overload‐ and prazosin‐induced angiogenesis. Modulation of capillary‐to‐fiber ratio (C:F), capillary domain area, capillary distribution (LogSD), citrate synthase activity (CS), hindlimb blood flow and muscle fatigue index after two weeks of muscle overload (A) or prazosin supplementation (B)

In this paper, we have highlighted the functional implications of differential angiogenic stimuli that harness morphologically distinct vessel growth (Figure 5). Specifically, overload‐driven angiogenesis improves functional capacity of the muscle through changes in local capillary supply, while angiogenesis induced by elevated shear stress lacks the specific adaptive remodelling required to improve fatigue resistance of skeletal muscle. Critically, we demonstrate that global capillary indices do not sufficiently predict functional capacity of skeletal muscle, and that local capillary supply should be quantified to improve assessment of the consequences for microvascular composition in adaptive remodelling, and aid characterization of disease prognosis.^30^, ^34^

The potent angiogenic potential of muscle overload and prazosin supplementation was reaffirmed in this study, with comparable increases in C:F to published overload^4^, ^20^ and prazosin^35^, ^36^ data. Similarly, the dynamic nature of the overload model has been highlighted, where a substantial hypertrophic response presents after the initial angiogenic burst.^4^, ^20^ This combined adaptive remodelling complicates interpretations of abluminal driven angiogenesis and its influence on function, as hypertrophy of muscle fibers confound area‐based indices of vascular supply, like that of CD.^34^, ^37^ However, our comprehensive overview and use of non‐scalar indices challenges the concept that more is better because, despite similar C:F changes to those observed with prazosin treatment, only overload improved fatigue resistance. Quantification of *in situ* vascular function demonstrated that prazosin supplementation, but not overload,^19^ significantly altered vascular tone by enhancing resting hindlimb blood flow.^36^ This increase in resting flow was not functionally relevant to muscle performance, as a similar arterial blood flow during stimulation existed between groups. Therefore, absolute functional hyperemia as measured by feed artery blood flow does not determine differences in fatigue resistance in these models, which instead are associated with targeted adjustments in microvascular topology within the muscle.

Through a novel combination of non‐scalar indices and mathematical modelling, we sought to characterize the coordinated angiogenic response and identify the driving mechanism for improved functional capacity.^13^, ^34^, ^38^ Indeed, following muscle overload there was a subtle shift in local capillary indices to a more homogeneous distribution with a significantly reduced supply area, indicative of an angiogenic response at the local tissue level coordinated to better match O_2_ supply and demand.^34^ Decreased CS activity reduced oxidative demand of the skeletal muscle, and consequently increased tissue PO_2_ in OV7. Decreased CS activity is indicative of either a decreased mitochondrial volume or reduced cristae density,^9^ and we speculate that this acute, reactive response may prevent excess oxygen delivery associated with increased capillarity, prior to the dynamic hypertrophy response elicited after 14 days of overload.^20^, ^24^, ^39^ This is consistent with the recovery of CS activity after 14 days of longitudinal stretch and corroborated by the restoration of modelled PO_2_. These time‐dependent adaptations may emphasise supporting tissue oxygenation prior to optimization of tissue function (such as a shift to slower phenotype seen after three weeks of muscle overload, *unpublished data*). Conversely, prazosin treatment failed to significantly affect the local capillary distribution (domain area and LogSD), corroborated by an unaltered tissue PO_2_ profile. The lack of influence on CS activity and predicted PO_2_ (associated with altered oxidative demand) indicates that the elevated microvascular supply following non‐physiological hyperemia appears to lack the necessary feedback control from the tissue to direct neovascularization that augments fatigue resistance.

As elevated shear stress associated with functional hyperemia evoked during endurance training clearly recruits an angiogenic response commensurate with the increased metabolic demand,^40^ this strongly indicates the absence of necessary feedback control elements in prazosin treatment. Our data may therefore explain why previous studies utilizing α‐adrenergic blockers to drive angiogenesis have seen only limited functional benefits. Terazosin supplementation in healthy humans has been used to increase skeletal muscle microvasculature, but with only a subtle improvement in tissue oxygen extraction.^41^ This may be in part due to a lack of improved local capillary supply,^30^, ^41^ associated with a stochastic angiogenic response. Similarly, prazosin treatment in a diabetic rodent model proved to be less effective than exercise in restoring impaired glucose homeostasis, emphasizing that the lack of specific tissue feedback hinders adaptive remodelling.^42^ Interestingly, glucose uptake is improved by prazosin‐mediated vessel proliferation in a healthy rat model,^43^ indicating that disease and the consequent extent of the existing capillary bed (NB. capillary rarefaction is typically observed in diabetes^42^) may determine the functional change in response to prazosin administration. While both prazosin and exercise promote angiogenesis,^30^, ^41^ pharmacological‐driven angiogenesis seems to lack the required control mechanisms present during exercise needed to coordinate adaptive remodelling, and why the combination of prazosin with exercise training proved to have an additive benefit to microvascular expansion and metabolic regulation.^42^ Recent experimental work further supports the importance of local feedback to drive improvements in muscle performance: microsphere‐induced vascular rarefaction led to greater capillary supply area, with a detrimental effect on fatigue resistance.^4^ These effects were alleviated with mechanical overload through a targeted distribution of neovasculature, detectable through improved local capillary distribution.

The divergent functional response to different angiogenic stimuli highlights the potential pitfall in pharmacologically induced vascular expansion. Of course, such interpretation comes with the caveat that this experimental work used healthy, young animals; contrasting results may be found after pharmacological expansion of the capillary bed in muscle that exhibits a compromised microcirculation due to diseases like COPD,^44^ heart failure^6^ or spinal cord injury.^45^ In this case, even stochastic vessel growth might prove beneficial in replacing otherwise lost capillaries. In conclusion, our data suggest that in the context of skeletal muscle microvascular supply, more does not necessarily mean better, and that the spatial distribution of vascular supply is a key determinant of functional capacity.

### 4.1 Perspective

Skeletal muscle microvascular rarefaction occurs in many diseases including COPD, heart failure and SCI, but optimal angiotherapy remains unclear. This research identified the critical importance of local capillary indices in predicting skeletal muscle performance, and how these should be considered when characterizing the effects of disease on skeletal muscle, and consequently the efficacy of rehabilitation angiotherapies. We may then be able to appropriately characterize dysfunction and develop targeted interventions. Although pharmacologically induced shear stress in healthy tissue was shown to be ineffective for restoring muscle performance, there remains a possibility that stochastic capillary growth in instances of severe microvascular rarefaction may offer therapeutic benefits.

## CONFLICT OF INTEREST

None declared.

## AUTHOR CONTRIBUTIONS

RWPK completed histological staining, analyses, *P*O_2_ modelling and drafted the manuscript. PGT completed surgical work, *in situ* experiments, statistical analyses and drafted the manuscript. NED completed the citrate synthase assay. AAS assisted with *P*O_2_ modelling. SE conceived the research, assisted experimental work and helped draft the manuscript.

## ETHICAL STATEMENT

We confirm that we have read the Journal's position on issues involved in ethical publication and affirm that this report is consistent with those guidelines.

## Data Availability

The datasets used and/or analyzed during the current study are available from the corresponding author on reasonable request.
